# Disturbance and distribution gradients influence resource availability and feeding behaviours in corallivore fishes following a warm-water anomaly

**DOI:** 10.1038/s41598-021-03061-w

**Published:** 2021-12-08

**Authors:** Chancey MacDonald, Hudson T. Pinheiro, Bart Shepherd, Tyler A. Y. Phelps, Luiz A. Rocha

**Affiliations:** 1 Department of Ichthyology, California Academy of Sciences, 55 Music Concourse Drive, San Francisco, CA 90118 USA; 2 Center of Marine Biology, University of São Paulo, Rod. Dr. Manoel Hipólito do Rego, km 131.5, São Sebastião, SP 11612-109 Brazil; 3 Steinhart Aquarium, California Academy of Sciences, 55 Music Concourse Drive, San Francisco, CA 90118 USA; 4 Department of Biology, San Francisco State University, 1600 Holloway Ave, San Francisco, CA 94132 USA

**Keywords:** Behavioural ecology, Climate-change ecology, Tropical ecology

## Abstract

Understanding interactions between spatial gradients in disturbances, species distributions and species’ resilience mechanisms is critical to identifying processes that mediate environmental change. On coral reefs, a global expansion of coral bleaching is likely to drive spatiotemporal pulses in resource quality for obligate coral associates. Using technical diving and statistical modelling we evaluated how depth gradients in coral distribution, coral bleaching, and competitor density interact with the quality, preference and use of coral resources by corallivore fishes immediately following a warm-water anomaly. Bleaching responses varied among coral genera and depths but attenuated substantially between 3 and 47 m for key prey genera (*Acropora* and *Pocillopora*). While total coral cover declined with depth, the cover of pigmented corals increased slightly. The abundances of three focal obligate-corallivore butterflyfish species also decreased with depth and were not related to spatial patterns in coral bleaching. Overall, all species selectively foraged on pigmented corals. However, the most abundant species avoided feeding on bleached corals more successfully in deeper waters, where bleaching prevalence and conspecific densities were lower. These results suggest that, as coral bleaching increases, energy trade-offs related to distributions and resource acquisition will vary with depth for some coral-associated species.

## Introduction

A key question in the Anthropocene is how ecosystems, communities, and populations respond to increased spatio-temporal variability in environmental and ecosystem states^1^. Warming of the atmosphere and oceans drives disturbances such as wildfire, drought, and coral bleaching that result in increasing biodiversity losses^2,3^. However, disturbance effects are rarely spatially uniform^4^ and often attenuate in severity and return frequency along environmental gradients^5–7^. Unequal disturbance impacts are reinforced by variability in the distributions and resilience mechanisms of species and their sub-populations^8^. Therefore, to predict species’ persistence outcomes it is first necessary to understand how spatial gradients in multiple environmental components interact with species’ ecologies^9^.

Behavioural mediation of ecological dynamics can strongly influence species’ responses to rapid environmental changes^10^. For example, behavioural flexibility can promote persistence under changed environmental conditions, through decisions such as where to move or what to eat^11,12^. Where resource degradation is spatially patchy, or attenuates along steep gradients, motile populations may be expected to spatially track key resources^13,14^. However, rapid or ephemeral degradation may promote flexibility in resource use as a better buffer of energetic losses and population declines, either via intensive foraging on degraded primary resources or through resource selection^15,16^. Therefore, understanding how gradients in environmental change and resource degradation interact with behavioural flexibility related to the access and utilization of quality resources is critical for assessing species’ futures in changing ecosystems.

Coral reefs are one such ecosystem experiencing strong global environmental pressures, the greatest of which is increased coral-bleaching frequency, driven by warming oceans (i.e., the stress-related expulsion of photosynthetic algal endosymbionts). Increasing atmospheric and oceanic temperatures mean annual bleaching is predicted in most tropical regions as early as 2030^3,17^. Bleaching events often result in broadscale coral loss and novel ecosystem states that restructure coral-associated assemblages^18–21^. However, bleaching does not always result in coral mortality. For example, after recent bleaching events, up to 60–98% of coral colonies across diverse global geographic locations have recovered^22–28^. Moreover, outcomes often vary among taxa and environmental conditions (e.g.^29^), and mortality rates can also decline with increasing water depth. For example, most coral genera and many species in the Indo-Pacific region exhibit depth-driven reductions in bleaching susceptibility^29–33^; but c.f.^34^. Therefore, increasingly frequent temperature-stressors are likely to result in increasingly ephemeral coral health conditions that may drive spatiotemporal pulses in resource quality for obligate coral associates. Correspondingly, direct behavioural responses during intra-bleaching periods - ~ 10–14 weeks from bleaching outset and preceding coral recovery or mortality^22^ - are likely to play increasingly important roles in the energetic budgets of coral-obligate organisms.

Among reef fishes, post-disturbance outcomes are often related to a species’ need and ability to behaviourally mediate resource declines^35^. The greatest losses frequently occur among species that have high intra-group competition for available resources, such as highly specialized, spatially restricted, coral-obligate species^21^. However, population attrition rates and lag times vary^21,35^ and it remains poorly understood how species may offset losses by using or avoiding bleached coral resources during intra-bleaching periods. Bleached corals may represent poor quality resources for associated species in several ways; such as disrupting crypsis-based predator avoidance in coral dwelling species^36^, nutritional intake, or decreasing visual acuity in polyp selection for corallivores^37^. Alternatively, stress-triggered secretions may provide carbon-rich food to corallivores adapted to scraping coral surfaces, rather than to biting polyps^38^, particularly during initial bleaching (see^39^). The ability or willingness to feed on bleached corals is likely to impact long-term population stability, as corallivore avoidance of bleached corals is linked with high attrition of abundance^21,39,40^ and local extinction^41^. Therefore, understanding species preferences and abilities to utilize or avoid bleached corals during rapid stress periods will help inform how species cope with increased resource pulses associated with human-induced ecological changes.

A key natural feature of coral reefs is the depth attenuation of many environmental processes (e.g. light and heat energy), organismal distributions, and their interactions^42,43^. However, SCUBA limitations mean detailed behavioural investigations are almost exclusively focused in shallow waters (but see^44–47^). Recent studies demonstrate that deep-resident corallivorous fishes can access greater total resources than their shallow-water counterparts, with fewer conspecifics, fewer competitive interactions and no apparent reductions in energy intake or body condition^45,46^. Therefore, parallel depth gradients in corallivore densities^47^ and coral bleaching intensity^29,30^ may result in greater relative future opportunities for deep-dwelling coral exploiters, despite lower resource availability^48,49^. However, it remains unknown how depth influences the use and avoidance of bleached coral resources. A particularly relevant question, therefore, is how concurrent depth gradients in coral densities, corallivore distributions and coral bleaching influence behavioural responses that may mediate energetic losses associated with resource declines on heat-stressed reefs.

Here, we utilized advanced technical diving operations to access shallow and upper-mesophotic coral ecosystems during a coral bleaching event related to a sustained thermal anomaly. We investigated depth-related patterns in coral-disturbance and the behavioural responses of corallivore fishes. We specifically hypothesized that: (1) The extent of coral bleaching will attenuate with depth and vary among coral genera; (2) The abundance of obligate coral feeding butterflyfishes will likely decline with depth, but may also be related to the availability of healthy (pigmented) corals if spatial resource tracking occurred; (3) Corallivorous fish will selectively feed on pigmented coral colonies and avoid bleached colonies at all depths, and (4) Depth-attenuation in heat-stress-related coral bleaching and competitor densities will result in corallivorous fish feeding proportionally more on pigmented corals at greater depths.

## Results

### Bleaching outcomes

**Figure 1:**
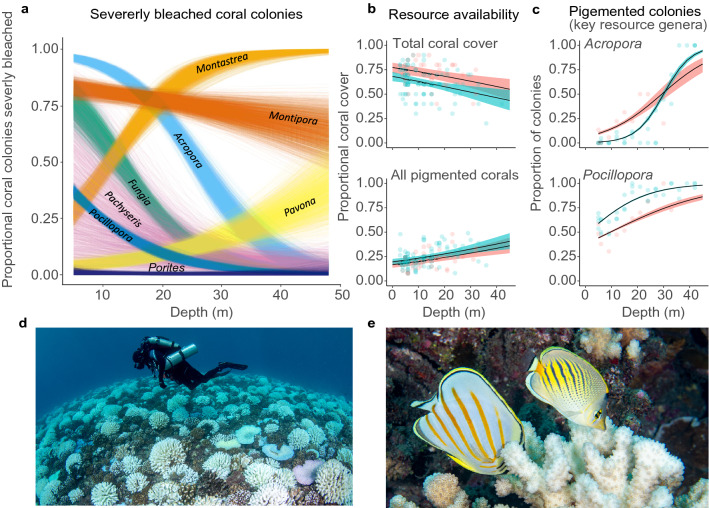
Differing levels of coral bleaching attenuation and health along a shallow to mesophotic depth gradient in Moorea, French Polynesia. (**a**) Variation in the proportion of severely bleached colonies (majority of colony white or with visible algae growing on recently bleached skeleton) among eight coral genera. (**b**) Matching site level depth variation in total coral cover (above) and cover of remaining pigmented corals (below). (**c**) The proportion of colonies remaining pigmented for the two primary prey genera of the focal fish species at the study location. (**d**) A research diver (Bart Shepherd) hovering over extensive coral bleaching during a decompression stop at a shallow study site. (**e**) Two of the focal fish species (Left: *Chaetodon ornatissimus;* Right: *Chaetodon pelewensis*) feeding on a bleached *Pocillopora* coral in shallow waters. Each line in (**a**) represents 1 of 2000 total draws in a Bayesian sampling model. In (**b**) and (**c**); red (site = Sofitel), blue (site = Rose Garden). Photos: Luiz Rocha.

Depth trends in bleaching responses (Fig. 1) varied among coral genera (Fig. 1a) and sites (Supplemental Fig. S3, Supplemental Table S1). Importantly, from a corallivore perspective, the proportion of pigmented colonies (i.e., not bleached) in the highly utilized coral genera *Acropora* and *Pocillopora* increased substantially with depth [depth trend (UCL, LCL); *Acropora: RG* = 0.0921 (0.077, 0.11), *Sf* = 0.19 (0.18, 0.20); *Pocillopora: RG* = 0.052 (0.045, 0.059), *Sf* = 0.090 (0.086, 0.095)] (Fig. 1c, Supplemental Table S2). Whereas an average of only 0–10% of *Acropora* colonies remained pigmented (level 1) at 5 m, ~ 75–90% were pigmented at 40 m. Among *Pocillopora* colonies, ~ 50–70% were pigmented at 5 m, compared to ~ 80–100% at 40 m, depending on site (Fig. 1c). Additionally, whilst total availability of corals declined with depth (i.e., coral cover, regardless of bleaching status) [depth effect: − 0.023 (− 0.036, − 0.010)]—with slightly lower coral cover at all depths within the Sofitel site [site effect: − 0.46 (− 0.73, − 0.20)], the remaining cover of pigmented corals increased with depth at both sites [depth = 0.023 (0.013, 0.033); site = 0.19 (− 0.01, 0.40)] (Fig. 1b).

### Spatial organization of corallivores

**Figure 2:**
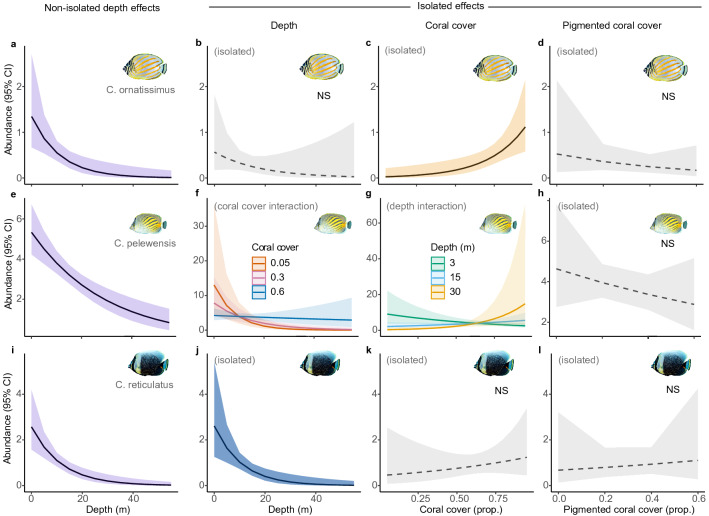
The distributions of three obligate coral feeding butterflyfishes (aligned in rows) in relation the combined and isolated effects of water depth (0–50 m), total coral cover (0–80%) and pigmented coral cover (0–60%). The left column (**a**,**e**,**i**) shows butterflyfish densities along the depth gradient, without conditioning for the effects of coral cover and pigmented corals. The second column (**b**,**f,j**) shows butterflyfish density along the same depth gradient, after conditioning for the effects of coral cover gradients. In this column, (**f**) shows a significant interaction between depth and total coral cover (i.e., regardless of bleaching condition), with the later held constant at 5% (red), 30% (pink), and 60% (blue). The third column (**c**,**g,k**) shows the effects of total coral cover isolated from depth and pigmented coral cover. In this column, (**g**) shows an interaction with depth held constant at shallow (3 m — green), intermediate (15 m — blue) and upper mesophotic (30 m — orange) depths. The final column (**d**,**h,l**) shows the effects of pigmented coral cover after conditioning on coral cover and depth effects. *NS* signifies non-supported hypotheses, which are further highlighted with dashed mean-effect lines and greyed-out confidence intervals. See Supplemental Fig. S7 for more information about conditioning on covariates to isolate main effects.

The abundance of all three fish species declined with depth (Fig. 2a,e,i) [*C. ornatissimus* -0.09 (−0.16, −0.04), R^2^ = 0.33; *C. pelewensis* −0.04 (−0.05, −0.02), R^2^ = 0.59; *C. reticulatus* −0.09 (−0.13, −0.05), R^2^ = 0.45]. However, when isolating the causal effects of total coral cover and pigmented coral cover from depth effects (see Supplemental Fig. S7), all three species had different distribution correlates. The distributions of *C. ornatissimus* were related solely to a positive association with higher total coral cover (irrespective of bleaching effects; Fig. 2c), but not the cover of pigmented corals (Fig. 2d) or to depth (Fig. 2b), outside of depth-effects on coral cover (i.e. coral cover had a direct effect, depth had an indirect effect, and pigmented coral cover had no effect) [isolated effects: R^2^ = 0.50; Depth −0.05 (−0.25, 0.2); Total coral cover 4.17 (1.40, 7.14); Pigmented coral cover −1.90 (−6.16, 2.49)]. The decline in abundance of *C. pelewensis* with depth (Fig. 2e) was also related to changes in total coral cover; however, in this case through an interaction of the two influences [Isolated effects: R^2^ = 0.57; Depth:Coral cover = 0.21 (0.07, 0.36)]. While more individuals occupied areas of high coral cover in deep water than in shallow water (Fig. 2f,g), *C. pelewensis* was most abundant in areas with the lowest coral cover. The relationship between the distribution of *C. pelewensis* and pigmented coral cover was not supported (Fig. 2h) [Depth:Pigmented coral cover = −0.18 (−0.42, 0.06)]. The strong effect of depth on the abundance of *C. reticulatus* (Fig. 2i,j) was isolated from effects of either total coral cover (Fig. 2k) or pigmented coral cover (Fig. 2l) [Isolated effects: R^2^ = 0.51; Depth = −0.09 (−0.15, −0.04); Depth:Coral cover = −0.014 (−0.36, 35); Coral cover = 1.27 (−2.13, 4.55; Depth:Pigmented coral = −0.21 (−0.86, 0.38); Pigmented coral = 3.28 (−2.46, 9.52)]. Notably, *C. pelewensis* was approximately twice as abundant at our study sites as *C. ornatissimus* [effect at 18 m depth = -2.10 (−2.68, −1.51)] and one and a half times more abundant than *C. reticulatus* [effect at 18 m depth = −1.45 (−1.04, −1.87)] (Supplemental Fig. S4).

### Feeding behaviour

#### Selectivity

**Figure 3:**
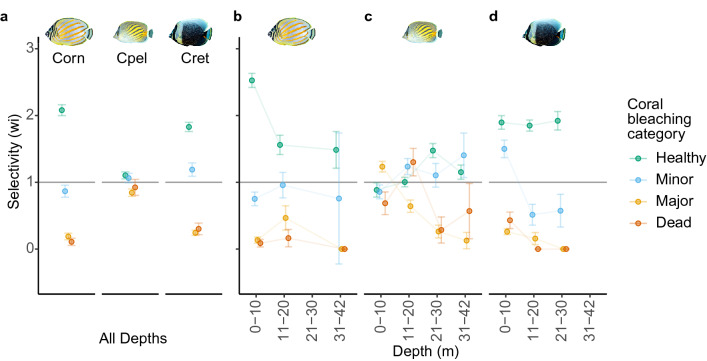
Feeding selectivity and avoidance behaviours in relation to four levels of coral bleaching, for three obligate corallivorous butterflyfishes in Mo’orea, French Polynesia. Feeding preferences across all depths for all three species (**a**) and contrasts of feeding preferences among depths for each species (**b**–**d**). Corn = *Chaetodon ornatissimus* (b), Cpel = *Chaetodon pelewensis* (c)*,* Cret = *Chaetodon reticulatus* (**d**). Resource selection measures (metric = $\hat{{\text{w}}}_{{\rm i}}$) with confidence intervals spanning values wholly > 1 indicate resource selectivity (i.e., resource use is high relative its environmental availability), values spanning wholly < 1 indicated resource avoidance, and values containing resource avoidance, and values containing 1 indicate resource use is proportional to availability.

All three focal fish species demonstrated selective feeding activity. All three species fed almost exclusively from hard corals (*C. ornatissimus* and *C. reticulatus* = 99% of bites; *C. pelewensis* = 98%) and chose to feed on pigmented corals overall, while actively avoiding strongly-bleached colonies (Fig. 3a; *C. ornatissimus,* Khi^2^L = 799.31, df = 3, p < 0.001; *C. pelewensis,* Khi^2^L = 33.21, df = 3, p < 0.001; *C. reticulatus,* Khi^2^L = 861, df = 3, p < 0.001). Selection for pigmented corals was strongest among *C. ornatissimus* [$\hat{{\text{w}}}_{{\rm i}}$ (LCL, UCL) = 2.11 (2.03, 2.20), p < 0.001] and *C. reticulatus* [$\hat{{\text{w}}}_{{\rm i}}$ = 1.82 (1.76, 1.90), p < 0.001]*,* which fed on pigmented corals at nearly twice the expected rate, given overall availability. Overall selection for pigmented corals was weaker among *C. pelewensis* [$\hat{{\text{w}}}_{{\rm i}}$ = 1.10 (1.05, 1.55), p < 0.001]*.* No species actively avoided feeding on corals with minor bleaching, with *C. reticulatus* selectively feeding on these mildly affected colonies [$\hat{{\text{w}}}_{{\rm i}}$ = 1.19 (1.09, 1.29), p < 0.001], while the other two species fed on them as much as expected given their availability [*C. ornatissimus*, $\hat{{\text{w}}}_{{\rm i}}$ = 0.90 (0.80, 1.00), p = 0.04; *C. pelewensis,* $\hat{{\text{w}}}_{{\rm i}}$ = 1.06 (0.99, 1.13), p = 0.08]. Interestingly, while both *C. ornatissimus* [$\hat{{\text{w}}}_{{\rm i}}$ = 0.10 (0.05, 0.15), p < 0.001] and *C. reticulatus* [$\hat{{\text{w}}}_{{\rm i}}$ = 0.30 (0.22, 0.39), p < 0.001] actively avoided feeding on recently dead colonies, overall, *C. pelewensis* did not [$\hat{{\text{w}}}_{{\rm i}}$ = 0.92 (0.80, 1.04), p = 0.211].

#### Depth influences on selectivity

Overall feeding selectivity patterns were generally stable with depth for two species, *C. ornatissimus* and *C. reticulatus*, but not for *C. pelewensis*. For *C. ornatissimus*, resource selectivity patterns were mostly constant along the depth gradient, although the level of selectivity for pigmented corals was 1.7 times greater in the shallowest 10 m compared to deeper depths (Fig. 3b). This was also the only depth at which *C. ornatissimus* actively avoided feeding on corals suffering minor bleaching [$\hat{{\text{w}}}_{{\rm i}}$ = 0.80 (0.70, 0.91), p < 0.001]. The feeding selectivity behaviour of *C. pelewensis* varied strongly with depth (Fig. 3c). For example, whereas *C. pelewensis* appeared to avoid feeding on pigmented colonies at 0–10 m [$\hat{{\text{w}}}_{{\rm i}}$ = 0.89 (0.78, 0.99), p = 0.036], at 11–20 m it fed on pigmented colonies in accordance with their availability [$\hat{{\text{w}}}_{{\rm i}}$ = 1.00 (0.93, 1.08), p = 0.92], and at depths > 20 m they were positively selected [21–30 m: $\hat{{\text{w}}}_{{\rm i}}$ = 1.48 (1.37, 1.58), p < 0.001; 31–42 m: $\hat{{\text{w}}}_{{\rm i}}$ = 1.51 (1.05, 1.26), p = 0.006]. A similar pattern of increased selectivity was evident among colonies with minor bleaching effects (Fig. 3c). Surprisingly, whereas *C. pelewensis* avoided feeding from recently dead and majorly bleached colonies in depths > 20 m (Fig. 3c), the species positively selected majorly bleached colonies at 0–10 m [$\hat{{\text{w}}}_{{\rm i}}$ = 1.48 (1.37, 1.58), p < 0.001] and dead colonies at 11–20 m [$\hat{{\text{w}}}_{{\rm i}}$ = 1.51 (1.05, 1.26), p = 0.006]. *Chaetodon reticulatus* consistently selected to feed on pigmented colonies at all depths (Fig. 3d). There was a strong depth-related decline in the level of selectivity for feeding on colonies with minor bleaching, where they were strongly selected for in waters less than 10 m depth and avoided at greater depths. The level of avoidance of recently dead and majorly bleached colonies also increased with depth for *C. reticulatus*.

#### Realized avoidance of bleached colonies

**Figure 4:**
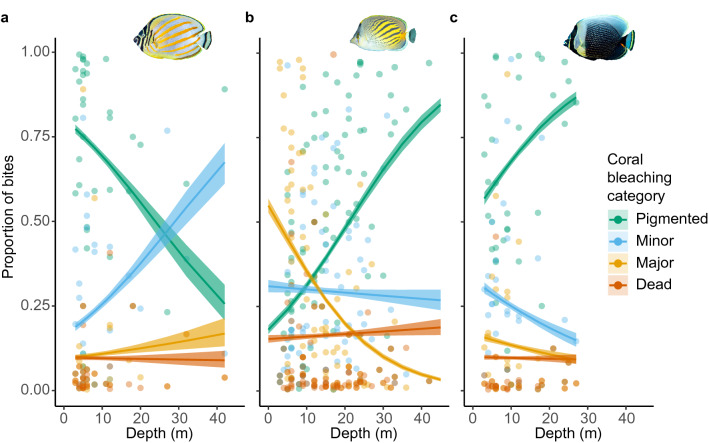
The proportion of bites taken from coral colonies of varying health, along a shallow-mesophotic depth gradient for three obligate corallivore butterflyfish species; (**a**) *Chaetodon* *ornatissimus*, (**b**) *Chaetodon* *pelewensis*, and (**c**) *Chaetodon* *reticulatus*.

Overall bite rates across all corals, regardless of bleaching, did not change with depth within any of the fish species [log–log scale model effect estimates: *C. ornatissimus* 0.01 (−0.06, 0.05), *C. pelewensis* −0.01 (−0.33, 0.30), *C. reticulatus* 0.25 (−0.75, 0.25); (Supplemental Fig. S5)]. However, the relative proportion of bites from colonies with different bleaching levels varied with depth for all three species (Fig. 4). Whereas *C. ornatissimus* fed proportionally less on pigmented colonies at deeper depths, the relative proportion of bites taken from pigmented corals at the deepest sample depths was between 50% higher than at 3 m for *C. reticulatus* and 370% higher for *C. pelewensis* [prop. of bites at 3 m and 40 m (95% CL), for *C. ornatissimus* 0.77 (0.76, 0.79) > 0.30 (0.21, 0.49); *C. pelewensis* 0.21 (0.20, 0.23) < 0.79 (0.77, 0.81); 3 m and 27 m for *C. reticulatus* 0.57 (0.56, 0.59) < 0.87 (0.85, 0.88)]. The proportion of bites *C. pelewensis* took from majorly bleached corals in shallow water was an order of magnitude higher than in deeper waters [3 m = 0.49 (0.47, 0.500), 40 m = 0.05 (0.04, 0.06)]. Interactions between total bites and the proportion of bites on pigmented colonies resulted in a higher overall feeding rate on pigmented corals at greater depths for *C. pelewensis* [0.09 (0.05, 0.14)] and equal overall feeding rates for both *C. ornatissimus* [log–log: −0.21 (−0.60, 0.18)] and *C. reticulatus* [−0.02 (−0.32, 0.30); Supplemental Fig. S6].

**Figure 5:**
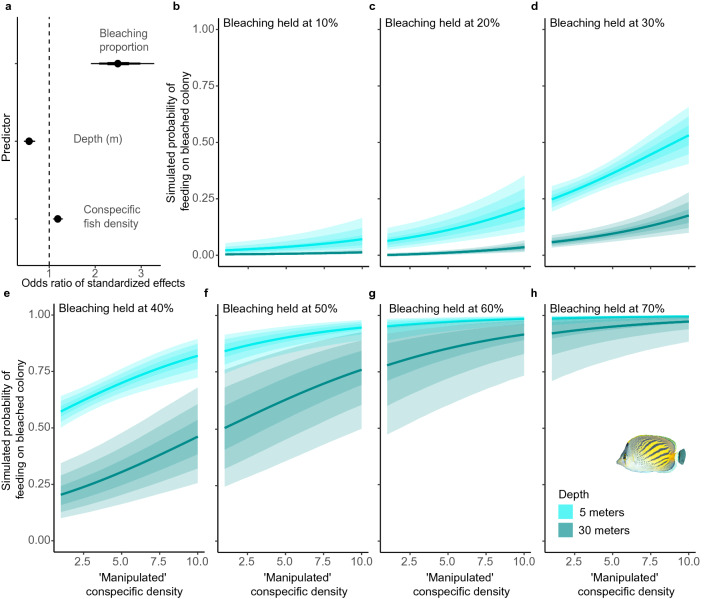
(**a**) Counterfactual plots showing the predicted effects of increasing conspecific density (1–10 conspecific individuals 40 m^−2^) on the probability of *C. pelewensis* feeding on a bleached coral colony (in any given bite) at each of two depths (5 m, 30 m) when the proportion of bleached coral colonies is held at each of seven fixed values (10–70%). (**a**) The odds ratios of effects for the three (standardized) predictors used in the model that the counter factual plots are built from. In (**b-h**), the gradated fills represent the 60%, 80% and 95% compatibility intervals (posterior mass) for each prediction. In (**a**), these same three intervals are shown with decreasing thickness of the three stacked error bars for each predictor. An odds ratio of 1 (indicated in (**a**) by the vertical dashed line) indicates no change in the odds of feeding on a bleached coral as the value of the predictor increases.

The odds of the abundant *C. pelewensis* feeding on a bleached coral in any given bite (Fig. 5) was influenced most strongly by the proportion of available colonies bleached, but also additionally by depth and conspecific density (Fig. 5a). Counterfactual plots estimated that manipulated conspecific densities would most strongly influence the probability of feeding on bleached colonies in shallow water (5 m) when 30–40% of colonies were bleached (Fig. 5d,e) and in deep water (30 m) when 40–50% of colonies were bleached (Fig. 5e,f). At these bleaching levels, adding one conspecific competitor was predicted to have approximately equal effect to increasing the proportion of bleached colonies by 1%. For example, at 30% bleaching (Fig. 5d), the probability of feeding on bleached corals in the shallows was ~ 0.25 with one conspecific per 40m^2^, which was the roughly equivalent to the probability for ten conspecifics with 20% bleaching (Fig. 5c). Additionally, with ten conspecifics at 30% bleaching (Fig. 5d) the probability (~ 0.55) was equivalent to having one conspecific competitor at 40% bleaching (Fig. 5e). The model also predicted that a bleaching prevalence of 30–40% would result in the greatest between-depth separation in the probability of feeding on bleached colonies. Again, at 30% bleaching (Fig. 5d), estimates of feeding probability for bleached colonies in deep water ranged from < 0.10 with one conspecific competitor to ~ 0.20 with ten conspecifics. When bleaching prevalence was below 20%, conspecific density was predicted to have little effect on feeding probabilities at 30 m (Fig. 5b,c). Finally, bleaching prevalence beyond 50% resulted in decreasing model support for differences in the probability of feeding on bleached colonies, either among depths or with changes in conspecific density.

## Discussion

As earth moves further into the Anthropocene, coral reef futures are unlikely to be spatially uniform. Here, we provide evidence that interacting spatial dynamics among resource availability, competition and disturbance can result in some resource-obligate species having greater opportunities to avoid degraded resources at deeper depths and at lower densities; even when species distributions do not spatially match rapid resource changes. While obligate corallivores actively avoided feeding on bleached corals, instead favouring pigmented corals, parallel attenuation of disturbance and competitor density gradients meant the remaining cover of pigmented corals and the ability to realize these feeding preferences increased with depth for two of the three species tested; despite a natural attenuation of pre-disturbance resource availability*.* Additionally, depth and total coral cover, regardless of bleaching condition, were better predictors of fish distributions than pigmented coral cover, suggesting that there was little to no immediate spatial organization of corallivore populations away from bleached corals.

The overall selection of pigmented coral food sources in this study suggests that the increasing frequency of bleaching events and recent broad documentation of bleaching recovery on tropical reefs may result in novel resource seasonality, emerging behavioural challenges, and increased energetic costs for corallivores (e.g.^17,41,50^). For example, heavy bleaching of preferred corals is related to global changes in butterflyfish competition interactions^50^ and the local extinction of a behaviourally inflexible corallivorous filefish^41^. Additionally, the two corallivorous butterflyfish species that have been shown to avoid bleached coral resources in previous small manipulation studies^39,40^ have among the top three attrition rates in response to coral losses, globally^21^.

The lack of evidence for strong resource tracking, via spatial matching to areas with greater cover of pigmented corals, suggests that established territories or home ranges are maintained or not dramatically altered during the initial phases of bleaching events. This may further suggest that spatial asymmetry in degradation pulses could result in some deep-resident corallivore species having prior-residency advantages^47^. Coral degradation is known to result in displacement of coral-dwelling fishes and spatial expansion of feeding territories among obligate corallivores^47,51–53^. Additionally, global-scale breakdowns of competition hierarchies in response to coral mortality^50^ and high mobility in larger non-territorial corallivore species are also expected to result in spatial restructuring^47^. In fact, spatial reorganization in response to changed resource availability is common among a wide range of motile foragers^14,15^. However, local corallivore abundances often remain stable for long periods following disturbances—albeit masking sometimes substantial sublethal effects^54,55^. The lack of spatial association with even total coral cover in *C. reticulatus* suggests some species may not associate strongly with total resource densities in the first place or that initial stages of spatial reorganization may have been underway. The costs of giving up established feeding territories for strictly resource-associated species can be high, particularly when resource reduction is rapid or alternative resources are previously occupied, as is the case among mice^56^. A previous experiment has demonstrated vertical territorial migration in resource-specialised corallivorous butterflyfishes, following localised resource losses, but only where established conspecific competitors have been removed^47^. It is unknown yet, whether increased disturbance frequencies will result in broadscale disruptions^50^ along the depth gradient, of the high interspecific aggression and intraspecific dominance hierarchies within corallivorous fishes^57^. It is further unknown whether this may result in the future numerical dominance of a few species, or even an opportunity to shift dominance hierarchies toward species that more readily occupy deeper reef habitats.

The strongest depth-related change in realized feeding behaviours in our study occurred in the abundant *C. pelewensis,* which fed proportionally more on pigmented coral colonies than bleached colonies in deep water, and whose probability of feeding on pigmented colonies also decreased with increasing conspecific densities. Our data support a density-mediated scenario for this species, where higher shallow-water abundances may have disrupted bleached-coral avoidance. A likely mechanism is increased competitive exclusion from pigmented corals, as disturbance can rapidly alter resource-consumer density ratios and competition dynamics^50^—though this was not directly tested. Dietary inflexibility among coral species or genera, may have also influenced this non-avoidance of bleached corals in shallow waters^41^. While genera-level feeding data was not recorded in this study, previous data from Mo’orea^58^ shows that *C. pelewensis* targets *Pocillopora* in a high percentage of bites in the region (~ 70%); however, it is not substantially more or less restricted in the number of coral genera it targets overall, compared to the other two focal species here (Supplemental Table S3). Interestingly, the mucus feeding *C. ornatissimus* demonstrated the most consistent avoidance of bleached corals and the highest selection of pigmented corals in shallow waters. This may suggest that mucus releases from bleaching corals do not provide substantive energy sources for corallivores; however; this may also be reflective of an ability to selectively target genera such as *Porites* and *Montipora*^58^ that are less palatable to many polyp-feeding corallivores and less susceptible to bleaching^22^. Variable responses among the three focal fish species here, demonstrate that differing abilities to utilise ecological flexibility are likely to result in varying levels of future risk. Our results therefore demonstrate that disruptions in stable food acquisition and energy trade-offs during future recurrent bleaching events will likely be non-uniform with depth and differ among species.

While coral bleaching and coral mortality can reach deeper waters, with sometimes catastrophic effects^34^, there is growing documentation of reduced bleaching susceptibility and mortality at depth among most coral genera and many broadly distributed species^29–32^. However, depth-attenuation of bleaching is unlikely a panacea for coral reefs^42^ and it is important to note the sometimes-variable attenuation effects among years, locations, and local oceanographic processes^59–61^. Bleaching impacts in our study also appeared to vary in relation to previously documented differences in natural inter- and intra-genera susceptibility, natural depth distributions and localized shading^29,62–65^. For example, bleaching prevalence in two genera, *Montastrea* and *Pavona*, increased with depth overall and Porites colonies showed almost no bleaching. While these genera were represented by few colonies overall, this is an unexpected trend and casual field observations suggest this is likely related to intra-generic species turnover along the depth gradient. Such variability in bleaching outcomes among coral genera is also likely to play a role in response variability among corallivores, which vary in coral preference and the ability to utilise dietary flexibility following resource loss. Importantly, the depth-mediated bleaching of coral genera widely preferred by obligate corallivores (most so by those with low dietary flexibility) observed here has been recorded in diverse locations across the broader Indo-Pacific^29,30,32^. This suggests that any energetic benefits sustained by deep-dwelling coral associates may be relatively widespread.

Depth-related attenuation in the bleaching of key coral resources and selective feeding on pigmented coral colonies by obligate corallivores resulted in increases in the relative proportion of bites taken from pigmented coral colonies at deeper depths for two of the three focal species, following a warm-water stress event. Because a large proportion of coral obligate populations currently intersect with the worst bleaching effects in shallow coral reefs, depth-related ecological dynamics are likely to play increasingly important roles in resource provision and energetic buffers for population maintenance and trophic energy transfers on coral reefs. The costs of resource and disturbance dynamics for foragers are often mediated by strong environmental gradients and effects can differ among trait groups and species^66,67^. Here we also show that sub-populations of some species that do not rapidly track spatial distributions to match spatiotemporal resource changes can continue to preferentially utilize non-degraded resources where disturbances attenuate along steep environmental gradients.

We propose that frequently recurrent heat stress will establish pulse resource disturbances on coral reefs and some coral associated species may exploit short-term energetic refuges at deeper portions of their ranges. Interestingly, previous studies have shown that despite lower population numbers at depth, deep-dwelling obligate corallivores have few apparent energetic disadvantages^45,46^. However, overall population sizes of coral associated organisms are likely to decline along with prey availability in shallow waters and the level to which depth can buffer the long-term effects of coral declines on tropical reefs will only become fully apparent through continued detailed investigations of the strong ecological dynamics at play along this steep environmental gradient.

## Methods

The study took place in Mo’orea, French Polynesia during May 2019, a period immediately after a warm-water anomaly of > 1 °C SST that lasted approximately six weeks^68^ (Supplemental Fig. S1). Sampling was conducted at two sites (Rose Gardens [RG] and Sofitel [Sf]). During the sampling period, water temperature generally decreased with depth, dropping from a maximum of ~ 28.3 °C in shallow depths to a minimum of ~ 24 °C at mesophotic depths (Supplemental Fig. S1).

To assess variation in coral bleaching immediately after the warm-water anomaly, we recorded the status of each coral colony from eight focal genera, within 49 depth-stratified plots between 3 and 42 m across the two sites. A total of 5,510 coral colonies were assessed from the eight focal genera: *Acropora, Fungia, Montastrea, Montipora, Pachyseris, Pavona, Pocillopora,* and *Porites.* Each plot was approximately 2 m^2^ and bleaching status was recorded on a four-point ordinal scale: (1) Pigmented = no visible lightening or tissue loss, (2) Minor bleaching = partial bleaching or lightening of colony, (3) Major bleaching = majority of colony white, (4) Recently dead = new algal growth visible on recently exposed skeleton. At the same time, we visually estimated total coral cover (irrespective of bleaching condition) within each plot (to the nearest 5%) and later estimated the cover of pigmented corals by multiplying the coral cover of each plot by the proportion of colonies that were not bleached or dead at the time of the surveys.

Butterflyfish abundance was recorded from 60 replicate in situ visual censuses (20 m × 2 m) by divers using closed circuit rebreathers, following^69^. The transects were distributed haphazardly across 25 depths (from 4 to 55 m), covering the same areas and period of coral and fish-behaviour assessments. We assessed the proportional feeding effort of the three most abundant obligate corallivores butterflyfish species (family: Chaetodontidae; *Chaetodon ornatissimus, C. pelewensis,* and *C. reticulatus*) on corals along the same depth gradient (Supplemental Fig. S2). We counted the number of bites taken from corals in each bleaching category by the three fish species, in replicate feeding observations between 3–42 m (*C. ornatissimus,* n = 35 observations and 1162 bites*; C. pelewensis,* n = 87 observations and 2713 bites*; C. reticulatus,* n = 37 observations and 1617 bites). The fish were observed for 5-min periods, with the average depth recorded. While all three species are obligate corallivores, *C. ornatissimus* feeds mostly by scraping coral tissue and ingesting mucus, while the other two feed directly on polyps^58^. Additionally, all three species are considered to have pair-forming, territorial social systems, though potentially with a decreasing strength of fidelity from *C. pelewensis* to *C. reticulatus*^70^. Territories are maintained via behavioural interactions that include inter- and intra- specific aggressions.

We focus aspects of our detailed bleaching results on *Acropora* and *Pocillopora* as they are preferred coral prey for obligate corallivores throughout the Indo-Pacific region, including for our focal fish species in Mo’orea; where all three species usually feed predominantly (51–68% of bites) on *Pocillopora* corals and also feed heavily from *Acropora* (Supplemental Table S3)^58^.

### Analyses

#### General modelling approach

We used R, version 3.5.2 for all analyses (references for statistical platforms and packages are presented in Supplemental Table S4). As a general procedure for analyses of depth patterns in response variables (each outlined in sections below), we used generalized linear models (glm) (Packages: ‘lme4’, ‘betareg’, ‘glmmTMB’, ‘brms’: Supplemental Table S4). We tested the predictive performance of candidate models for each response variable using Akaike’s information criteria (AIC) (‘MuMIn’), log-likelihood tests, and multi-metric model comparison function in ‘performance’, as appropriate. When using AIC, models with ΔAIC > 2 were considered to have suitably different predictive performance, otherwise fits were further compared with log-likelihood tests (using α = 0.05). Presented models had the highest predictive performance of plausible candidate models—i.e., the lowest AIC score, log-likelihoods with p < 0.05; or via multi-metric model comparison function in ‘performance’. All final models were considered suitable representations of the data after assessing distributions of model residuals and model dispersion parameters for non-bayesian glm (‘DHARMa’, ‘Betareg’) and a full range of Bayesian diagnostics (‘brms’, and ‘rstan’), as appropriate. The fit of predicted values, from modelled estimates, to raw data was assessed using R^2^ approximations for non-bayesian glm (‘performance’). Among-level differences in significant predictors were further analysed using post-hoc trends contrasts (‘emmeans’).

#### Bleaching outcomes and coral cover

Bleaching outcomes were tested with glms fit with candidate models using; (a) depth only, (b) with depth intercepts varying between locations, (c) with depth slopes and intercepts varying among locations. To test for variation in depth attenuation of bleaching impacts among coral genera*,* slopes and intercepts of depth trends in a glm of pooled bleaching categories (levels 2–4) were allowed to vary among sites and genera (i.e., a three-way covariate interaction model; ΔAIC > 1300). In order to visualize overall variation in depth attenuation patterns of severe bleaching (levels 3–4) among the focal coral genera across sites, we used a 1000-draw plot of a Bayesian binomial glm, built in ‘Rstan’. We additionally tested for depth variation in the availability (% cover) of total coral resources and of pigmented corals only, using beta-regression models in ‘betareg’. For total coral cover, the best predictive model used depth slopes with varying intercepts between sites (i.e., depth with site as an additive covariate, ΔAIC = 2.2), whereas for pigmented coral cover the site covariate did not improve model fits compared to depth (ΔAIC 1.18, p > 0.05). Additionally, we modelled depth influences on the proportion of colonies remaining pigmented (level 1) for two key coral genera for butterflyfishes (*Acropora* and *Pocillopora*), using betabinomial distributions and logit links, with site as an interacting covariate (ΔAIC > 200, ΔAIC > 800, respectively).

#### Spatial organisation of focal corallivore species

To investigate potential influences of coral bleaching on the spatial organisation of corallivorous butterflyfishes, we modelled abundance counts of each of three focal species against a small number of a-priori predictor variable combinations, using glm with *Poisson* distributions and logit links. We first ascertained the causal pathways among predictors (depth, total coral cover, pigmented coral cover) and the response variables (fish abundance) using Directed Acyclical Graphs (DAGs) and causal theory developed by Pearl et al.^71^. This allowed us to utilise statistical models that isolated the effects of the target predictor variables, through conditioning on causally related covariates. A substantive explanation and illustration of our use of this method of causal inference in model selection is presented in Supplemental Fig. S7 and its caption. Further technical detail is available in^71^, and pedagogical examples of its use are available in^72^. To outline the methodological outcomes utilized here; the distribution of each species was first modelled against depth only, this model captured all effects associated with changes in depth, as well as the effects of depth itself. Second, depth effects were isolated from effects of coral cover and pigmented coral cover by conditioning on (including in models) the covariates of total and bleached coral cover (see Supplemental Fig. S7). Thirdly the effects of total coral cover were isolated by conditioning on the effects of depth and pigmented corals. Finally, the effects of pigmented corals on fish distributions were isolated through modelling with the conditioning covariate influences of coral cover and depth. Because the effects of total coral cover and pigmented coral cover may vary along the depth gradient, models tested for interactions among depth and coral-cover-based covariates, which are reported when significant. In each model, statistics are only taken for the focal predictor, or for the focal predictor’s interaction with a covariate of interest. A description of the models used and their assessment via the multi-metric model comparison platform in ‘performance’ are available in Supplemental Table S5.

#### Feeding behaviour

##### Selectivity

We used Manly’s^73^ resource selection ratios in ‘adehabitatHS’ (Supplemental Table S3) to test for population level selectivity and avoidance (i.e., proportional resource use in relation to resource availability) of feeding on coral colonies in each bleaching level, for each of the three focal fish species, across all depths (all observations 0–42 m) and also within four sequential depth bins (0–10 m, 11–20 m, 21–30 m, 31–42 m). Selection ratios were calculated using the formula: $\hat{{\text{w}}}_{{\rm i}}$ = o_*i*_/a_*i,*_ where $\hat{{\text{w}}}_{{\rm i}}$ is the resource selection ratio for colonies with bleaching level *i*, o_*i*_ is the proportion of colonies with bleaching level *i* fed on and a_*i*_ is the proportion of colonies with bleaching level *i* available to be fed on. Proportional resource availability was calculated as the mean proportional cover of coral colonies from each bleaching category, recorded from all plots within the area of interest (i.e., mean of observations pooled across all depths, or within each depth bin. To account for multiple comparisons across the bleaching status categories, Bonferroni Z adjustments were used to calculate the upper (UCL) and lower (LCL) limits for 95% confidence intervals for each resource selection ratio. Resource selection measures (metric = $\hat{{\text{w}}}_{{\rm i}}$) with confidence intervals spanning values wholly > 1 indicate resource preference or selectivity, values spanning wholly < 1 indicated avoidance, and values containing 1 indicated random use (i.e., resource use is proportional to availability). Within-depth differences in feeding selectivity for corals within each bleaching status were assessed using log-likelihood Chi^2^ tests (metric = Khi^2^L).

##### Realized avoidance of bleached colonies

To test for depth related differences in overall bite rates we used log–log linear models (to meet assumptions of heteroscedasticity in model residuals) of bite rates on all colonies against observation depth, pooled across sites. To test for depth related variation in the proportional number of bites on colonies within each bleaching status level we modelled the proportion of total bites against observation depth, with bleaching status as a covariate, for each of the three fish species. For this, we used beta regression models in ‘betareg’, with a (*y × *(*n − *1*)* + 0.5)/*n* transformation to deal with 0, 1 inflation (where n is the sample size).

To disentangle the interacting influences of gradients in depth, the proportion of colonies bleached, and conspecific fish density (predictors) on the probability of corallivores feeding on bleached coral colonies (response variable) we used counterfactual plots of predictions based on estimates for the three predictors, developed in a Bayesian glm with a binomial distribution and logit link, using ‘brms’. Counterfactual plots explore the causal implications of manipulating one or more predictor variables, therefore showing the implied predictions for imaginary experiments^72^. We focussed this investigation on the abundant *C. pelewensis*. To utilise spatially comparative data from different collection depths among predictors and the response, we grouped mean estimates of the proportion of bleached coral colonies, fish density and feeding data from within 10 sequential 4 m depth bins (from 0 to 40 m), prior to modelling. We standardised the predictors (mean centred and scaled in relation to one standard deviation) to allow informative comparisons of effects among predictors on different scales. To keep prior model expectations within plausible parameters, we used normally distributed priors with means of 0 and a standard deviation of 1.5 for intercepts and a standard deviation of 0.5 for regression coefficients. We ran the model across four chains with 10,000 iterations per chain and confirmed model suitability using a full range of diagnostics (Pareto k: 95% < 0.5, 5% 0.5–0.7; Rhat: all = 1; Neff ratio: all > 0.9; Non-divergent trace plots; acf = low autocorrelation; density plots = unimodal and regular; trace plot = reasonable prior-predictive consolidation).

## Supplementary Information

Supplementary Information 1.

Supplementary Information 2.

Supplementary Information 3.

Supplementary Information 4.
