# Supplementary material for: Disturbance and distribution gradients influence resource availability and feeding behaviours in corallivore fishes following a warm-water anomaly
Source: Sci Rep. 2021 Dec 8;11:23656. doi: 10.1038/s41598-021-03061-w (PMC8654952; doi:10.1038/s41598-021-03061-w)
Supplement: Supplementary file 4 — Supplementary Information 4. [file 41598_2021_3061_MOESM4_ESM.docx]

**Supplemental Material for:**

**Disturbance and distribution gradients influence resource availability and feeding behaviours in corallivore fishes following a warm-water anomaly.**

Chancey MacDonald^1^, Hudson T. Pinheiro^1,2^, Bart Shepherd^3^, Tyler A. Y. Phelps^1,4^, Luiz A. Rocha^1^

^1^Department of Ichthyology, California Academy of Sciences, 55 Music Concourse Drive, San Francisco, CA, 90118, USA.

^2^Center of Marine Biology, University of São Paulo, Rod. Dr. Manoel Hipólito do Rego, km 131.5, São Sebastião, SP 11612-109, Brazil.

^3^Steinhart Aquarium, California Academy of Sciences, 55 Music Concourse Drive, San Francisco, CA, 90118, USA.

^4^Department of Biology, San Francisco State University, 1600 Holloway Ave, San Francisco, CA 94132 USA.


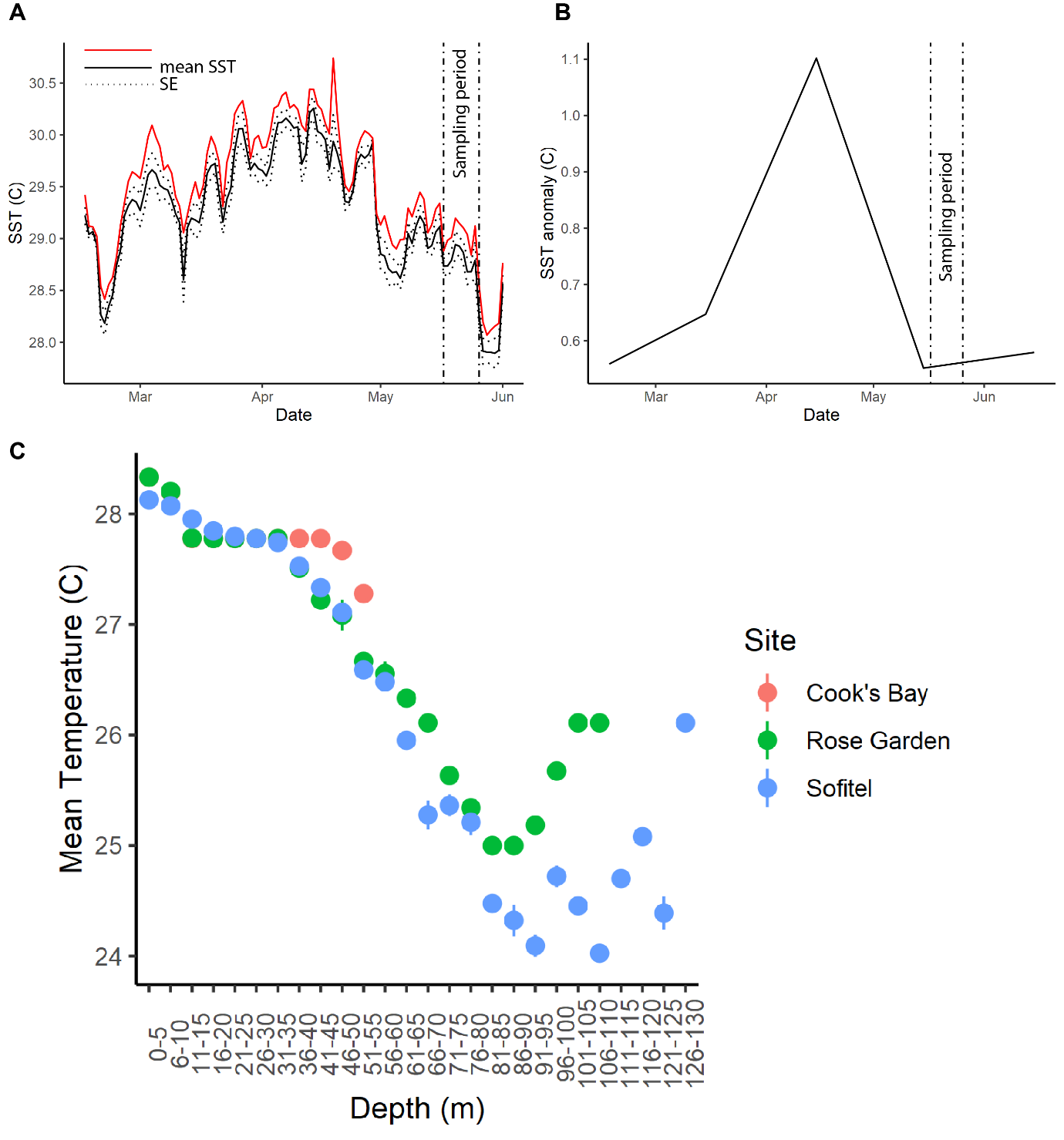


**Supplemental figure S1:** A sea surface temperature (SST) anomaly in Mo’orea, French Polynesia between February 15 and July 15, 2019, including the sampling period (shown in A and B). (A) Average and maximum sea surface temperatures for ocean areas directly surrounding Mo’orea (area shown in C). (B) SST anomalies for the same area. (C) Depth profiles for temperatures at each site during the sampling period. Error bars are 95% CI. Data for A-B are from the NOAA Coral Reef Watch global 5km [*CoralTemp* and Sea Surface Temperature (SST) Anomaly product](https://www.ospo.noaa.gov/Products/ocean/sst/anomaly/index.html). Data for C are from Sherwood ‘Pectral’ dive computers, used during this investigation.

 
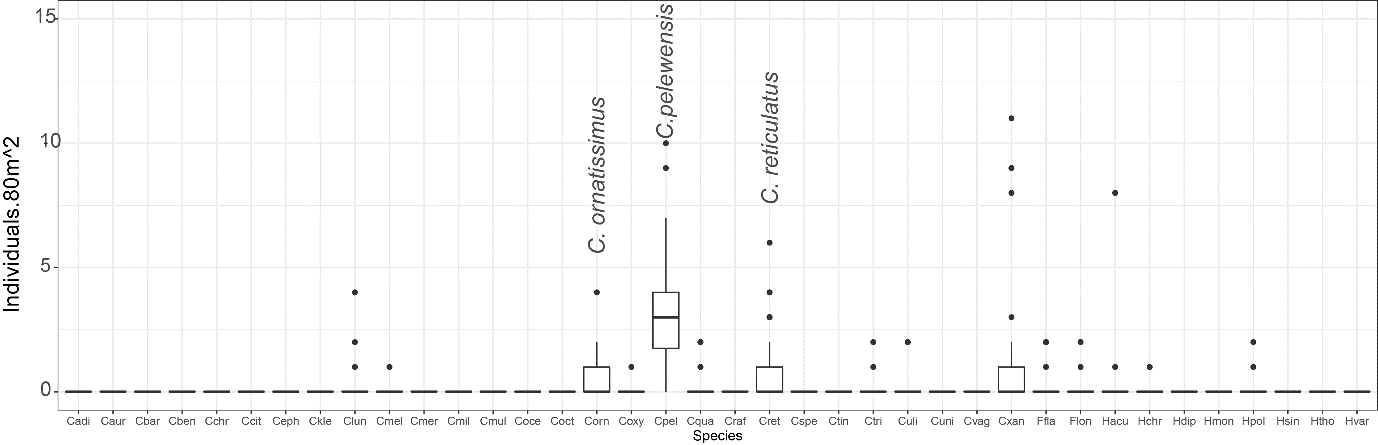


**Supplemental figure S2:** Abundances of species from the family *Chaetodontidae* in Mo’orea during the study period. The three focal species for our study are the three most abundant obligate corallivore species (Corn *= Chaetodon ornatissimus,* Cpel = *Chaetodon pelewensis,* Cret *= Chaetodon reticulatus).* Note: Cxan = *Chaetodon xanthurus* – anecdotally a facultative corallivore.

 
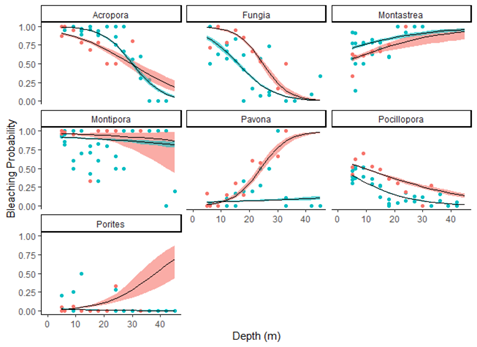


**Supplemental figure S3:** Differences in bleaching attenuation among genera and sites, along a shallow – mesophotic depth gradient in Mo’orea, French Polynesia.

**Supplemental table 1:** Depth trends and site contrasts for depth trends in the probability of coral bleaching among colonies from seven major coral genera.


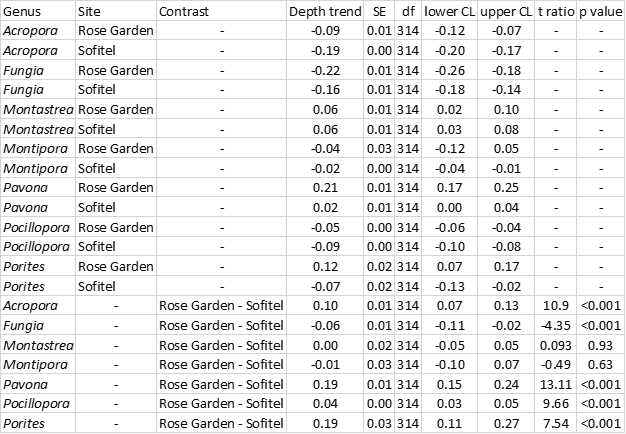


**Supplemental table 2:** Predicted probability of bleaching for *Acropora* and *Pocillopora* coral colonies at eight depths, including statistics for contrasting predictions between the two sites, Rose Garden and Sofitel. For coloured boxes, darker blues indicate deeper depths and deeper reds indicate a higher bleaching probability. Red dots indicate non-significance, orange dots indicate significance at alpha = 0.05 and green dots indicate significance at alpha = 0.001.

**
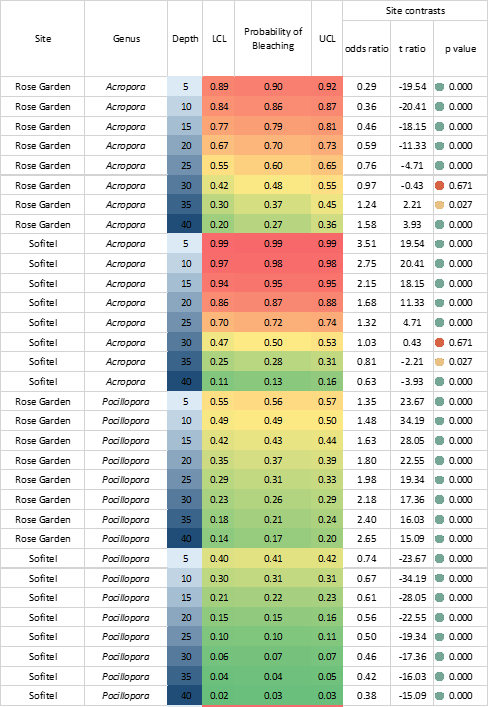
**

**
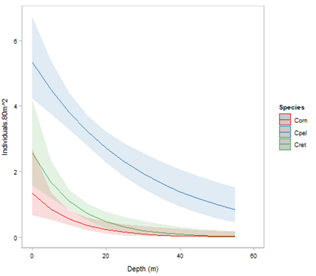
**

**Supplemental figure S4:** Abundance estimates for each of three focal obligate coral feeding *Chaetodon* species, between 0 and 55 m in Mo’orea, French Polynesia.  Corn (red) = *Chaetodon ornatissimus*, Cpel (blue) = *Chaetodon pelewensis*, Cret (green) = *Chaetodon reticulatus*.

 
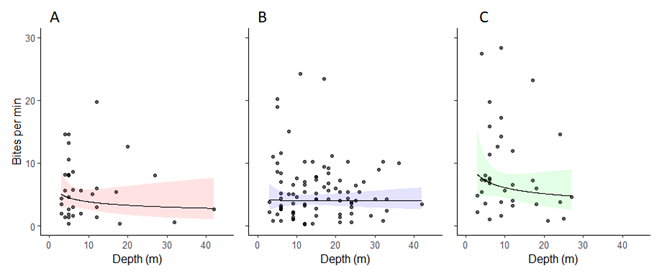


**Supplemental figure S5:** Overall bite rates along a depth gradient in Mo’orea, for each of three focal butterflyfish species. (A) *Chaetodon* *ornatissimus*. (B) *Chaetodon pelewensis* (C) *Chaetodon reticulatus*. Bands represent 95% confidence intervals.

 
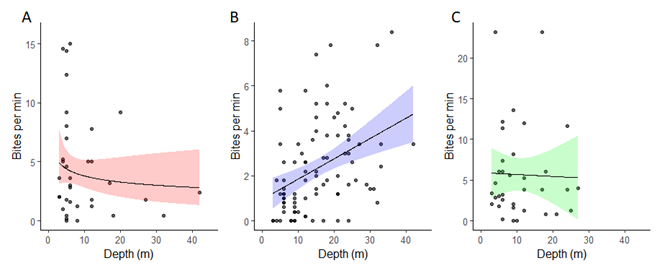


**Supplemental figure S6:** Bite rates on pigmented (non-bleached) coral colonies along a depth gradient in Mo’orea, for each of three focal butterflyfish species. (A) *Chaetodon* *ornatissimus*. (B) *Chaetodon pelewensis* (C) *Chaetodon reticulatus*. Bands represent 95% confidence intervals.

**Supplemental table 3:** Coral use by the focal fish species in Mo’orea. Data collected by Berumen and Pratchett, and published in Pratchett (2013)

**Supplemental table 4**: References for packages and platforms used for statistical analyses.

| **Package name** | **Reference** |
| --- | --- |
| R version 3.5.2 | R core team, 2020 |
| lme4 | Bates et al., 2015 |
| betareg | Cribari-Neto & Zeileis, 2010 |
| glmmTMB | Brooks et al., 2017 |
| brms | Bürkner, 2017 |
| MuMIn | Barton, 2009 |
| DHARMa | Hartig, 2020 |
| performance | Lüdecke et al., 2020 |
| emmeans | Lenth, 2020 |
| Rstan | Stan development team 2020 |
| adehabitatHS | Calenge, 2006 |

**
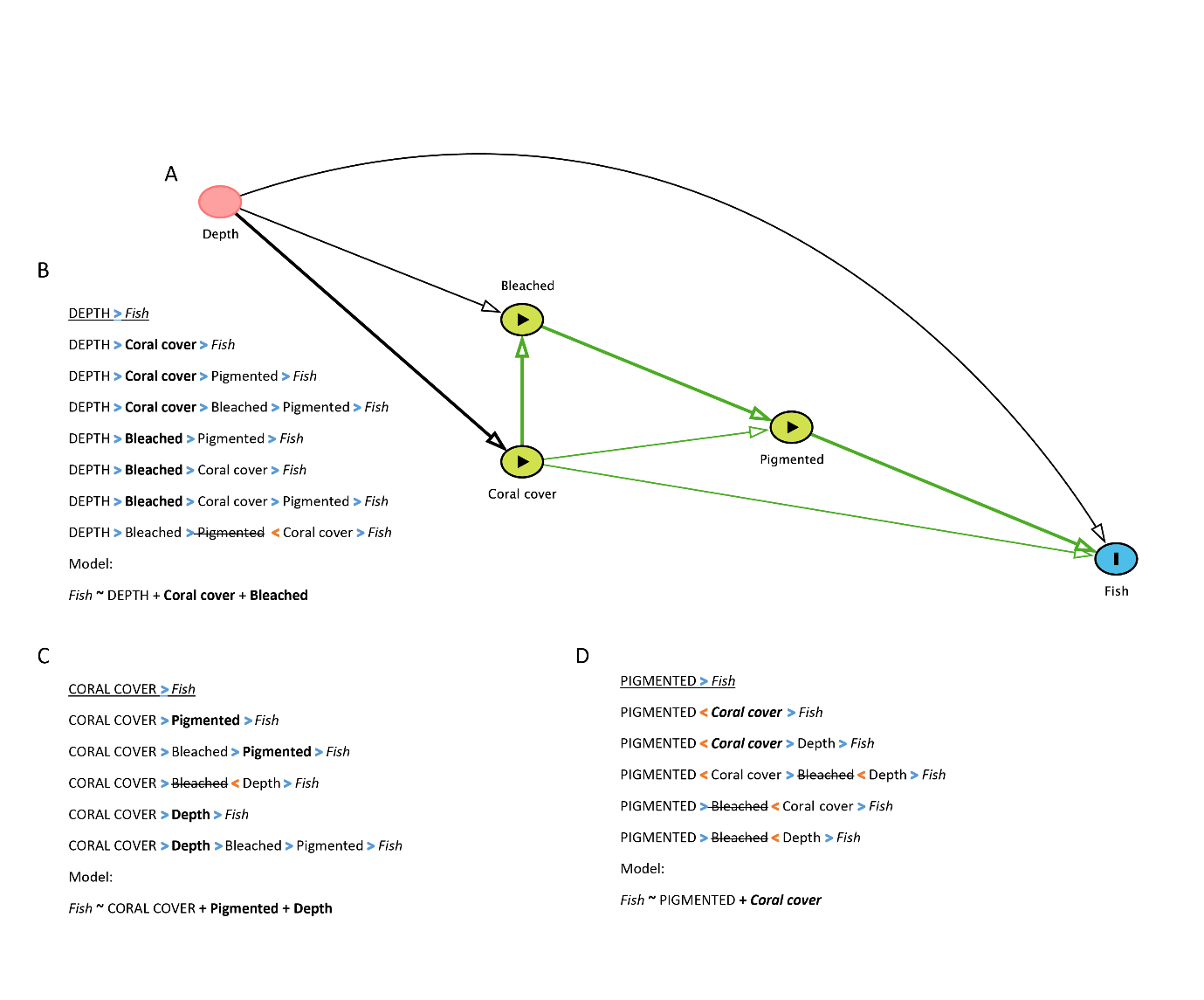
**

**Supplemental figure S7: The use of Directed Acyclical Graphs (DAGs) for inferring causality in statistical models.** Based on, Pearl et al. (2016) and McElreath (2020). The example here was used in this investigation to isolate the influences of depth, total coral cover, and pigmented coral cover on the distribution of butterflyfish abundances.

A Directed Acyclical Graph (DAG) **(A)** shows the causal relationships among covariates in a model of fish abundances and are used as an aid to construct adequate statistical models. Arrow directions show the causal relationships between pairs of predictor covariates, as well as covariates and the response variable. By using the following observations and rules, DAGs can better inform predictor selection in statistical models and strongly decrease spurious and masked relationships.

Guidelines for use:

1) Causality is directional, but information can flow in both directions, along connecting lines (i.e., regard arrows and lines for causality, disregard arrows for information flow in statistical models) For illustrative purposes, think of flipping the x and y axes in a correlation plot - regardless of direction a relationship between the two variables is present).

2) The goal of an using an adequate statistical model is reached by identify and isolating all possible information paths between the response variable and the predictor of interest (see all possible paths for **(B)** ‘Fish abundance ~ Depth’, **(C)** Fish abundance ~ Total Coral cover’, and **(D)** ‘Fish abundance ~ Pigmented coral cover’. Note: the key relationship is underlined for each model).

3) Arrows entering and leaving a covariate in the same direction (i.e. two arrows of the same colour in B-D) represent an information ‘pipe’ and a pipe must be blocked in order to isolate the influence of the predictor of interest (all caps in B-D) from other covariates. To do this in practice, at least one (but more is allowable) of the covariate predictors in a line of pipes must be included in the statistical model. In B-D, the first possible covariate predictor to include in each pipe is highlighted in bold.

4) Covariates with causal arrows leaving a covariate in opposite directions represent ‘Forks’ and (like pipes) these must also be included in the model to stop the flow of information leaks from covariates into the relationship between the predictor of interest and response variable.

5) Covariates with causal arrows entering a covariate in opposite directions represent a ‘confounder’. These paths are already closed, unless the confounder is included in the statistical model, in which case the path is then erroneously opened. For this reason, confounder covariates are represented in B-D by being struck out. They must not be included in the model.

5) Covariates in a path act in series. Therefore, only one break needs to be included in any one path; either the inclusion of a ‘pipe’ or ‘fork’ variable, or the exclusion of a ‘confounder’ variable. The final model (last line of B-D) therefore consists of the response variable (italics in B-D) modelled against the key response variable (all caps in B-D) with the minimum covariates necessary to block spurious relationships (Bold in B-D) and without covariates that would introduce masked relationships (i.e., no struck-out covariates in B-D).

**Supplemental table 5**: Model performance statistics used to select between additive and interaction models for the isolation of the effects of coral cover and pigmented coral cover on the distributions of the focal fish. Corn = *C. ornatissimus*, Cpel = *C. pelewensis,* Cret = *C. retrofasciatus*. Reported model for each model set is highlighted in bold.

…DC = Fish ~ DEPTH + Coral Cover + Bleached Coral Cover

…DCi = Fish ~ DEPTH * Coral Cover + Bleached Coral Cover

…MCC = Fish ~ CORAL COVER + Depth + Pigmented Coral Cover

…MCCi = Fish ~ CORAL COVER *Depth + Pigmented Coral Cover

…MLC = Fish ~ PIGMENTED CORAL COVER + Depth + Coral Cover

…MLCi = Fish ~ PIGMENTED CORAL COVER * Depth + Coral Cover

**References:**

 Barton, K. (2009) Mu-MIn: Multi-model inference. R Package Version 0.12.2/r18. [http://R-Forge.R-project.org/projects/mumin/](http://r-forge.r-project.org/projects/mumin/)

Bates, D., Mächler, M., Bolker, B., & Walker, S. (2014). Fitting linear mixed-effects models using lme4. arXiv preprint arXiv:1406.5823.

Bürkner, P. C. (2017). brms: An R Package for Bayesian Multilevel Models Using Stan. *Journal of Statistical Software*, 80(1), 1-28. doi:10.18637/jss.v080.i01

Calenge C (2006). “The package adehabitat for the R software: tool for the analysis of space and habitat use by animals.” *Ecological Modelling*, **197**, 1035.

Hartig, F. (2020). DHARMa: Residual Diagnostics for Hierarchical (Multi-Level / Mixed) Regression Models. R package version 0.2.7. [https://CRAN.R-project.org/package=DHARMa](https://cran.r-project.org/package=DHARMa)

Lenth R; (2020). emmeans: Estimated Marginal Means, aka Least-Squares Means. R package version 1.4.5. https://CRAN.R-project.org/package=emmeans

Lüdecke, D., Makowski, D., Waggoner P., & Patil I. (2020). Performance: Assessment of Regression Models Performance. R package version 0.4.5. [https://CRAN.R-project.org/package=performance](https://cran.r-project.org/package=performance)

McElreath, R. (2020). *Statistical rethinking: A Bayesian course with examples in R and Stan.* CRC press, Boca Raton, Florida.

Pearl, J; Madelyn, G; Nicholas, J. P. (2016) *Causal inference in statistics*. John Wiley and Sons, West Sussex

Pratchett, M. S. (2013). Feeding preferences and dietary specialization among obligate coral-feeding butterflyfishes. *Biology of butterflyfishes. CRC Press, Boca Raton, FL*, 140-179.
